# Increased Placental Cell Senescence and Oxidative Stress in Women with Pre-Eclampsia and Normotensive Post-Term Pregnancies

**DOI:** 10.3390/ijms22147295

**Published:** 2021-07-07

**Authors:** Paula J. Scaife, Amy Simpson, Lesia O. Kurlak, Louise V. Briggs, David S. Gardner, Fiona Broughton Pipkin, Carolyn J. P. Jones, Hiten D. Mistry

**Affiliations:** 1 Clinical, Metabolic and Molecular Physiology Research Group, University of Nottingham, Nottingham NG7 2RD, UK; paula.scaife@nottingham.ac.uk; 2 Department of Obstetrics & Gynaecology, University of Nottingham, Nottingham NG7 2RD, UK; amy.e.simpson98@gmail.com (A.S.); Fiona.broughton_pipkin@nottingham.ac.uk (F.B.P.); 3 School of Veterinary Medicine and Science, University of Nottingham, Nottingham NG7 2RD, UK; lesia.kurlak@nottingham.ac.uk (L.O.K.); David.gardner@nottingham.ac.uk (D.S.G.); 4 School of Engineering, University of Nottingham, Nottingham NG7 2RD, UK; louise.briggs@nottingham.ac.uk; 5 Maternal & Fetal Health Research Centre, Manchester Academic Health Science Centre, University of Manchester, Manchester M13 9PL, UK; carolyn.jones@manchester.ac.uk; 6 Department of Women and Children’s Health, School of Life Course Sciences, King’s College London, London SE5 9NU, UK

**Keywords:** hypertension in pregnancy, angiogenesis, endothelial function, oxidative stress, antioxidants, post-maturity, senescence

## Abstract

Up to 11% of pregnancies extend to post-term with adverse obstetric events linked to pregnancies over 42 weeks. Oxidative stress and senescence (cells stop growing and dividing by irreversibly arresting their cell cycle and gradually ageing) can result in diminished cell function. There are no detailed studies of placental cell senescence markers across a range of gestational ages, although increased levels have been linked to pre-eclampsia before full term. This study aimed to determine placental senescence and oxidative markers across a range of gestational ages in women with uncomplicated pregnancies and those with a diagnosis of pre-eclampsia. Placentae were obtained from 37 women with uncomplicated pregnancies of 37–42 weeks and from 13 cases of pre-eclampsia of 31^+2^–41^+2^ weeks. The expression of markers of senescence, oxidative stress, and antioxidant defence (tumour suppressor protein p16^INK4a^, kinase inhibitor p21, interleukin-6 (IL-6), NADPH oxidase 4 (NOX4), glutathione peroxidases 1, 3, and 4 (GPx1, GPx3, and GPx4), placental growth factor (PlGF), and soluble fms-like tyrosine kinase-1 (sFlt-1)) genes was measured (quantitative real-time PCR). Protein abundance of p16^INK4a^, IL-6, NOX4, 8-hydroxy-2′-deoxy-guanosine (8-OHdG), and PlGF was assessed by immunocytochemistry. Placental NOX4 protein was higher in post-term than term deliveries and further increased by pre-eclampsia (*p* < 0.05 for all). P21 expression was higher in post-term placentae (*p* = 0.012) and in pre-eclampsia (*p* = 0.04), compared to term. Placental P16^INK4a^ protein expression was increased post-term, compared to term (*p* = 0.01). In normotensive women, gestational age at delivery was negatively associated with GPx4 and PlGF (mRNA and protein) (*p* < 0.05 for all), whereas a positive correlation was seen with placental P21, NOX4, and P16^INK4a^ (*p* < 0.05 for all) expression. Markers of placental oxidative stress and senescence appear to increase as gestational age increases, with antioxidant defences diminishing concomitantly. These observations increase our understanding of placental health and may contribute to assessment of the optimal gestational age for delivery.

## 1. Introduction

The human placenta stops growing at ~90% of full term (~36 weeks of gestation), unlike that of other mammalian species, but the fetus continues to grow, which would presumably “stress” even a normal placenta. This feature is assumed to have evolved in parallel with upright posture and the necessary development of a very muscular uterus, delaying delivery. Ageing is a process that causes a deterioration in function at the cellular, tissue, and organ level, leading to individuals being more susceptible to disease. Short chromosomal telomeres, as well as the partial or complete insufficiency of the telomerase enzyme, have been linked to diseases caused by ageing [1]. Telomeres are protective caps made of nucleoprotein molecules located at the end of chromosomes and are necessary for protection against breaks at DNA ends, fusion of chromosome ends, and chromosome degradation [1]. Telomeres are shortened with each cell division. The rate at which this occurs is accelerated by certain stressors, such as oxidative stress [1]. Eventually, telomeres reach a dangerously short length, which initiates the process of cellular senescence, through which cells irreversibly stop growing and dividing by arresting their cell cycle and gradually ageing (becoming ‘senescent’) [2,3].

Prolonged pregnancy (also known as post-term pregnancy), is defined by the World Health Organisation as “the end of gestation at ≥42 completed weeks of gestation, measured from the first day of the last menstrual period and based on a 28 day cycle” [4]. Adverse obstetric events have been linked with pregnancies that last longer than 42 weeks, including an increased frequency of foetal death and raised risk of foetal malnutrition, intrauterine foetal hypoxia, irregular nonstress tests, respiratory distress, oligohydramnios, delivery via Caesarean section, and stillbirth [5,6]. Furthermore, long-term health problems in the child have also been associated with pregnancies extending over 42 weeks [5].

In the developed world, up to 11% of pregnancies extend to post-term. However, this figure varies significantly between different countries with further disparity between low- and middle-income countries [5]. In the UK, a woman who has not spontaneously delivered by the start of 41 weeks is offered an induction of labour. The induction is performed between 41^+0^ and 42^+0^ weeks to avoid the associated risks of prolonged pregnancy [7].

The main risk factors associated with prolonged pregnancy are genetic, the mother having been born post-term herself, previous history of prolonged pregnancy, primiparity, and obesity [8]. Moreover, a Swedish study reported that one-quarter of the risk arises from the fetal genetic background, and another quarter arises from the mother [9].

Some of the signs associated with prolonged pregnancy are reduced fetal movements, a reduced volume of amniotic fluid, and meconium-stained amniotic fluid after the membranes have ruptured [10]. A seminal study conducted by Jones and Fox in 1978 investigated the structural changes in placentae after prolonged pregnancy and revealed that most of these placentae show morphological irregularities. A possible decline in trophoblastic cell function was postulated [11].

A study by Maiti et al. concluded that placental cells start to age dramatically from 37 weeks of gestation, with oxidative stress rising as gestational age increases [12]. Other studies suggest that premature placental ageing may contribute to placental dysfunction. This could be the cause of many placenta-related pathologies, such as pre-eclampsia [13], a multisystem disorder affecting up to 5% of pregnant women. It is one of the leading causes of maternal and perinatal mortality and morbidity, especially when it occurs before 34 weeks of gestation [14]. It is characterised by de novo hypertension together with evidence of endothelial cell damage and/or significant proteinuria in the absence of urinary tract infection [15]. Pre-eclampsia is associated with shallow placentation, inadequate remodelling of the uterine vasculature, and consequent oxidative stress; antioxidant defences are inadequate, and the synthesis of a variety of angiogenic and growth factors is perturbed [16].

Senescence stressors ultimately activate the P53 and/or cyclin-dependent kinase inhibitor 2A (P16^INK4a^) pathways and secondary induction of cyclin-dependent kinase inhibitor (P21) [2]. Furthermore, interleukin-6 (IL-6) depletion is correlated with reduced formation of senescence-associated heterochromatin foci (SAHF) and, thus, can also be used as a surrogate marker of senescence [17].

In summary, oxidative stress in combination with cell senescence can cause extensive tissue damage, leading to accelerated cellular ageing. We hypothesised that extended, ‘post-term’ pregnancies and gestational disease such as pre-eclampsia both increase oxidative stress and cell senescence in placentae, relative to healthy term controls. To investigate this, we examined the mRNA expression and protein abundance of a panel of markers of cell senescence (P16^INK4α^, P21, and IL-6), oxidative stress (NADPH oxidase 4 (NOX4), 8-Oxo-2′-deoxyguanosine (8-OHdg), a marker of DNA modification), antioxidant defence (glutathione peroxidases (GPx)), and placental function (PlGF and sFlt-1) in normotensive control and post-term placenta, as well as from women with pre-eclampsia.

## 2. Results

### 2.1. Participants

Baseline demographic and pregnancy outcome data are presented in Table 1. As can be seen, by definition, women who had pre-eclampsia had significantly higher blood pressures (*p* < 0.05) and significant proteinuria. They also delivered earlier, and their babies’ birthweights were lower. Overall, the groups were matched for maternal age, BMI, and parity.

### 2.2. Gene Expression

**Figure 1:**
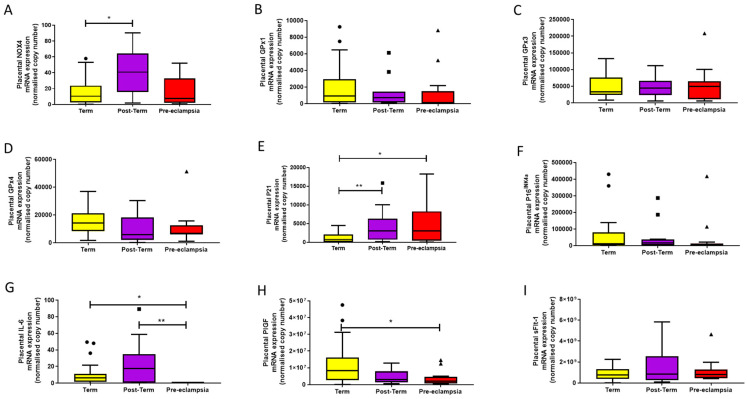
mRNA expression assessed by quantitative reverse-transcription PCR of (**A**) NADPH oxidase (NOX4), (**B**–**D**) glutathione peroxidases 1, 3, and 4 (GPx1, 3, and 4), respectively, (**E**) cyclin-dependent kinase inhibitor (P21), (**F**) cyclin-dependent kinase inhibitor 2A (P16^INK4α^), (**G**) interleukin-6 (IL-6), (**H**) placental growth factor (PlGF), and (**I**) soluble fms-like tyrosine kinase-1 (sFlt-1) in placentae from term normotensive (37–40 + 6 weeks; *n* = 26), post-term normotensive (41–42 weeks; *n* = 11), and women who had pre-eclampsia (*n* = 13). Data are presented as median [IQR]; * *p* < 0.05, ** *p* < 0.005.

Considering markers of oxidative stress, expression of NOX4 was present in all samples (Figure 1A). NOX4 expression was increased post-term, compared to placentae from term deliveries (*p* = 0.013). For all GPxs, no differences were observed between groups (*p* > 0.05 for all), although there was a trend towards lower GPx4 expression in both post-term and pre-eclampsia groups. When further sub-grouped by gestational age, we found significantly higher GPx4 gene expression in the 37–39^+0^-week placentae (median [IQR]: 17,765 [13,366, 25,101], normalised copy number) compared to the 40–40^+6^-week (8729 [2992, 13,823]; *p* < 0.01) and 41–42-week placentae (5739.1 [2160, 18,130]; *p* = 0.04), as well as in pre-eclampsia (*p* = 0.04).

All markers of senescence were expressed (Figure 1E–G) in every group. Placental P21 expression was raised in post-term deliveries (*p* = 0.012), as well as in women with pre-eclampsia (*p* = 0.04), compared to term samples. For IL-6, women with pre-eclampsia had a higher placental expression compared to both term (*p* = 0.031) and post-term (*p* = 0.008); no differences were seen between term and post-term samples (*p* > 0.05). No differences were observed between groups for P16^INK4α^.

We also examined expression of the placental functional markers, PlGF and sFlt-1 (Figure 1H,I). PlGF expression was lower in placentae from women with pre-eclampsia, compared to term (*p* = 0.008), but not post-term samples (*p* >0.05); smaller differences between term and post-term expression did not reach statistical significance (*p* = 0.08). No differences were seen between groups for sFlt-1 expression (*p* > 0.05).

Lastly, when considering any impact of gestational age at delivery in only the normotensive women, negative associations were observed with placental GPx4 (*r* = −0.405; *p* = 0.012) and PlGF (*r* = −0.388; *p* = 0.021), whereas a positive correlation was seen with P21 (*r* = 0.324; *p* = 0.044).

### 2.3. Protein Expression

**Figure 2:**
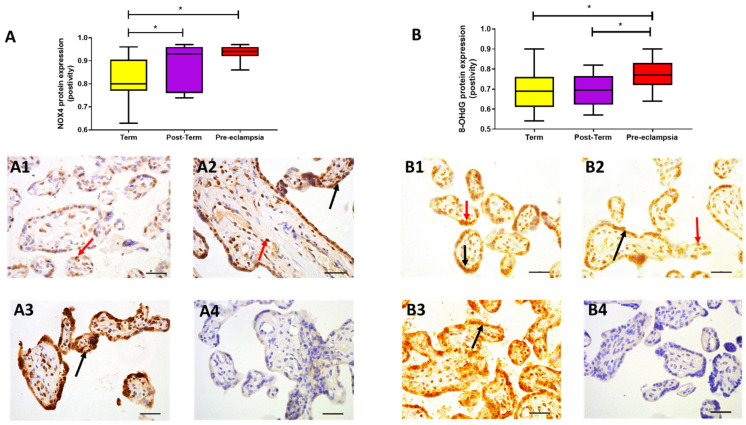
Protein expression assessed by immunohistochemistry. (**A**) NADPH oxidase (NOX4) and (**B**) 8-hydroxy-2′-deoxy-guanosine (8-OHdG) in placentae from term normotensive (37–40 + 6 weeks; *n* = 26), post-term normotensive (41–42 weeks; *n* = 11), and women who had pre-eclampsia, delivered at 31 + 2 to 41 + 2 weeks gestation (*n* = 13). Data are presented as median [IQR]; * *p* < 0.05. Photomicrographs show typical examples of immunostaining in (**A1**,**B1**) term, (**A2**,**B2**) post-term, (**A3**,**B3**) pre-eclampsia, and (**A4**,**B4**) IgG negative control. Positive protein expression appears brown and is localised mainly to the syncytiotrophoblast (black arrows) but is also evident in the nuclei (red arrows); scale bar = 100 μm.

NOX4 protein expression was confirmed in all placental samples analysed, with staining localised within nuclei and syncytiotrophoblast (Figure 2A). As with gene expression, NOX4 protein expression was higher in both post-term (0.95 [0.79, 0.97]; *p* = 0.017) and pre-eclampsia samples (0.94 [0.92, 0.96]; *p* < 0.0001; Figure 2A), compared to term samples (median [IQR]: 0.80 [0.77, 0.90] positivity), with more uniform, high expression observed in pre-eclampsia (*p* > 0.05). Placental expression of 8-OHdG was localised mainly within the nuclei with some weak cytoplasmic staining (Figure 2B) and was highest in women with pre-eclampsia (median [IQR]: 0.77 [0.72, 0.83] positivity), compared to both term (0.68 [0.61, 0.76]; *p* = 0.015) and post-term (0.70 [0.63, 0.76]; *p* = 0.021) samples (Figure 2B); similar expression was observed between term and post-term (*p* > 0.05).

**Figure 3:**
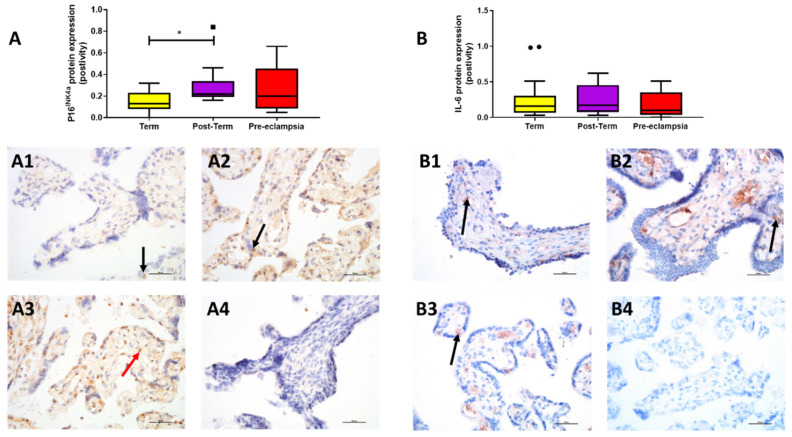
Protein expression assessed by immunohistochemistry. (**A**) P16^INK4a^ and (**B**) interleukin-6 (IL-6) in placentae from term normotensive (37–40 + 6 weeks; *n* = 26), post-term normotensive (41–42 weeks; *n* = 11), and women who had pre-eclampsia (*n* = 13). Data are presented as median [IQR]; * *p* < 0.05. Photomicrographs show typical examples of immunostaining in (**A1**,**B1**) term, (**A2**,**B2**) post-term, (**A3**,**B3**) pre-eclampsia and (**A4**,**B4**) IgG negative control. Positive protein expression of P16^INK4a^ appears brown and is localised mainly to the syncytiotrophoblast (black arrow) with some stromal cell staining (red arrow); that of interleukin 6 is found mainly in endothelial cells (arrows); scale bar = 100 μm.

P16^INK4a^ expression differed between groups (*p* = 0.036). Expression was higher in post-term (0.22 [0.20, 0.32]) compared to term (0.13 [0.09, 0.23]; *p* = 0.011) and was found mainly in the endothelium; however, placentae from women with pre-eclampsia exhibited similar expression (0.20 [0.09, 0.40]; *p* > 0.05; Figure 3A). IL-6 localised mainly to the endothelium of the villi and overall expression was low; expression did not differ between groups (*p* > 0.05; Figure 3B).

**Figure 4:**
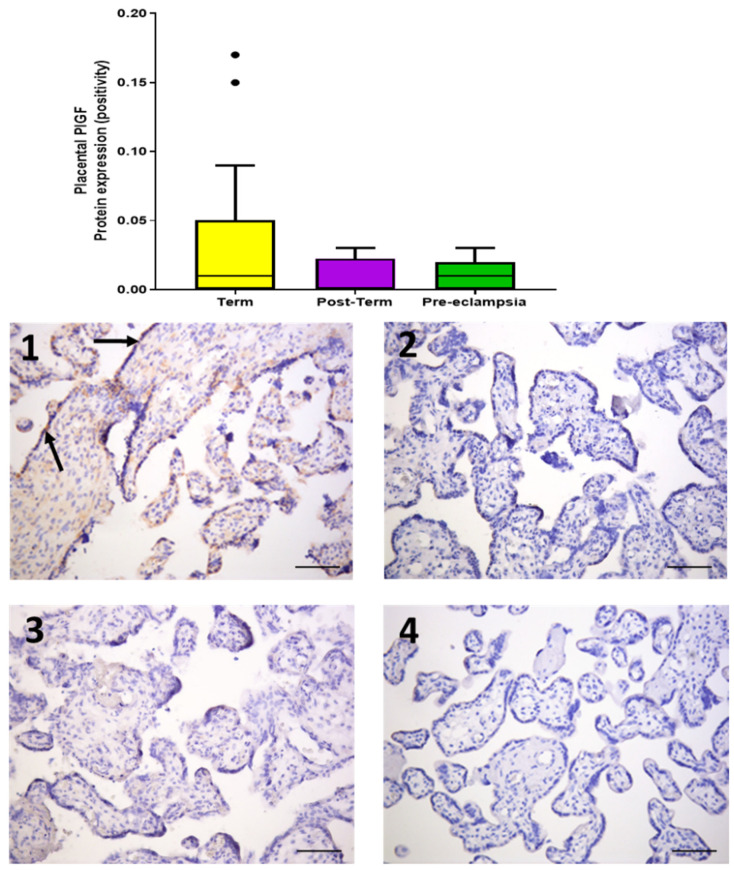
Protein expression of placental growth factor (PlGF) assessed by immunohistochemistry in placentae from term normotensive (37–40 + 6 weeks; *n* = 26), post-term normotensive (41–42 weeks; *n* = 11), and women who had pre-eclampsia (*n* = 13). Data are presented as median [IQR]. Photomicrographs show typical examples of immunostaining in (**1**) term, (**2**) post-term, (**3**) pre-eclampsia, and (**4**) IgG negative control. Positive protein expression is weak and appears brown, localised mainly to the syncytiotrophoblast (black arrows); scale bar = 100 μm.

PlGF expression was weak and localised to the syncytiotrophoblast layer (Figure 4) and, although levels did not differ statistically between groups (*p* > 0.05), there was a trend towards lower expression in post-term (0.0024 [0.0013, 0.023]) and placentae from women with pre-eclampsia (0.0069 [0.0039, 0.019]), compared to term (0.0073 [0.0026, 0.045]) (Figure 4).

**Figure 5:**
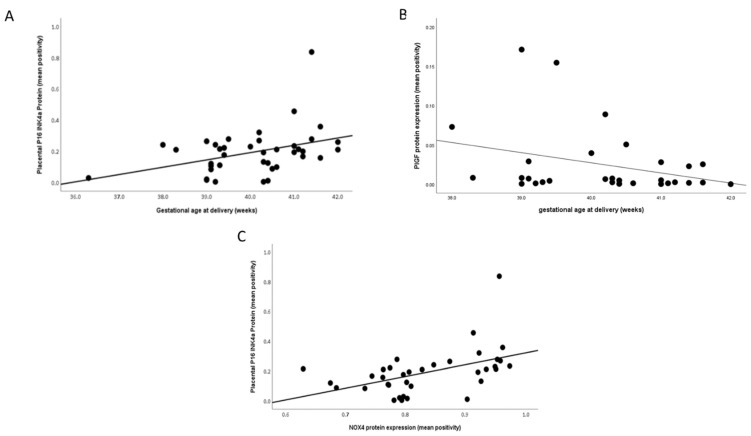
Scatter plot illustrations showing correlations between gestational age at delivery and (**A**) P16INK4a (*r* = 0.331; *p* = 0.04) and (**B**) placental growth factor (PlGF; *r* = −0.367; *p* = 0.046); (**C**) between P16^INK4a^ and NADPH oxidase 4 (NOX4; *r* = 0.537; *p* = 0.001) in normotensive samples only.

Again, when considering the impact of gestational age at delivery in only the normotensive women, a positive association was evident with placental P16^INK4a^ expression (*r* = 0.331; *p* = 0.04; Figure 5A). In contrast, a negative correlation was seen with PlGF expression (*r* = −0.367; *p* = 0.046; Figure 5B). In addition, placental expression of P16^INK4a^ and NOX4 were positively correlated (*r* = 0.537; *p* = 0.001; Figure 5C).

## 3. Discussion

Features of placental ageing may play a role in the morbidity associated with prolonged gestation. After 39 weeks of pregnancy, the fetal death rate rises fourfold [18,19]. Maiti et al. studied placentae from three groups: term (39 weeks), late term (>41 weeks), and idiopathic stillbirths; they found increased aldehyde oxidase 1 expression (a mediator of placental ageing), DNA/RNA and lipid oxidation, lysosomes situated perinuclearly and basally as opposed to apically when localised with lysosome-associated membrane protein 2 (LAMP2), and bigger autophagosomes suggestive of inhibition of function in later-term placentae [12]. Towards the end of pregnancy and amplified during the post-term period, the foetal demands for oxygen and nutrients outstrip the placenta’s ability to supply, thus leading to increased oxidative stress and subsequent deterioration of the placenta, including marked senescence [20].

We have now shown increased cell senescence and oxidative stress not only in placentae from normotensive women who delivered post term but also from women who developed pre-eclampsia, when compared to normotensive women delivering at term. The fact that increased gene and protein expression of NOX4, one of the sources of cellular reactive oxygen species (ROS), was evident in both post-term and pre-eclamptic groups implies that these placentae are under significant oxidative stress. Whilst this is well known in pre-eclampsia [21], this is the first detailed report in normotensive post-term placentae.

In this study, 8-OHdG protein expression was only raised in the placentae from women who had pre-eclampsia in concordance with others [13], reflecting oxidative DNA damage. However, one other study has shown increased 8-OHdG expression in post-term placentae, using a different technique, specifically, counting the number of positively stained nuclei [12]. Nevertheless, increased levels of oxidative stress cause damage to DNA, proteins, and lipids in placental tissue, which may manifest as another form of accelerated placental ageing. The antioxidant GPX genes also showed a trend towards reduced levels in the post-term and pre-eclamptic tissue. There were, however, no significant differences between groups in the protein expression of the cytokine IL-6, despite gene expression being raised in the pre-eclampsia group. A possible reason for this is that IL-6 is downstream of senescence, not an initiator of it [22]. Thus, placental senescence in post-term pregnancy does not activate this particular inflammatory cascade or has not been around long enough to do so.

In this study, increased protein expression of the senescence marker P16^INK4a^ was observed in post-term and pre-eclamptic placental tissue. The p16/pRb and p53/p21 pathways are reported to be activated in various cell lines in response to stimuli that induce irreversible cellular senescence [23,24]. Cell fusion, an essential physiological process to establish and expand the syncytiotrophoblast, has been recognised to be a further trigger of cell senescence, and the syncytiotrophoblast features characteristics of senescent cells including the biomarker senescence-associated beta-galactosidase (SA-β-gal), together with high expression of the cyclin kinases inhibitors p16, p21, and p53 [25], which regulate cell-cycle progression at G_1_ and S phase. It must be noted that syncytiotrophoblast senescence is a normal physiological phenomenon, which progresses as pregnancy advances [26]. However, there is increasing evidence to suggest that, when physiological senescence is accelerated, it results in placental and clinical pathology. Cindrova-Davies et al. reported placental P21 protein expression to be higher in post-term and pre-eclampsia samples [13]. Moreover, increased placental or trophoblast senescence has already been demonstrated in terms of telomere shortening, aggregation, or other measures of telomere dysfunction, both in pre-eclampsia and in normotensive women with foetal growth restriction [27,28,29,30,31].

Our data also demonstrated that mRNA and protein expressions of the proangiogenic growth factor PlGF were lower in placentae from women with pre-eclampsia and post-term samples, although the latter did not achieve statistical significance. It is known that pre-eclampsia is partly mediated by dysfunctional syncytiotrophoblast, and PlGF (as a marker for syncytiotrophoblast health) has appeared as a good marker for early-onset pre-eclampsia [32]. However, this is not the case in late-onset pre-eclampsia occurring towards term. Interestingly, in uncomplicated normotensive pregnancy, circulating PlGF concentrations rise steadily, peaking around 30 weeks, and then fall [32]. This suggests that the syncytiotrophoblast becomes increasingly stressed for the last 8–10 weeks of pregnancy [33] and possibly beyond. Further supporting data show that pO_2_ measurements of maternal intervillous and umbilical venous and arterial bloods decline in the third trimester, with slowing placental growth [34]. These data collectively suggest that normotensive women also have placentae with syncytiotrophoblast stress both at term and post-term [33].

When defining ‘term’ pregnancy according to the American College of Obstetricians and Gynaecologists, the neonatal outcome measures on delivery vary greatly between delivery at 37^+0^ and 42^+0^ weeks [35]; therefore, the importance of accurately establishing the gestational age cannot be overemphasised. Nonetheless, there are many countries in which mothers are unable to access ultrasound scans and do not have verifiable measurements of gestational age, meaning that the actual rate of post-term births in such countries may be higher than that officially recorded [5].

In this study we wished both to identify whether placentae from post-term pregnancies showed evidence of increasing senescence and to determine whether placentae from pre-eclamptic women showed accelerated senescence earlier in pregnancy. Both post-term pregnancy and pre-eclampsia are associated with increased risk of sudden fetal death, presumably consequent on placental failure. This is a preliminary study; hence, we chose to investigate well-established markers of several different aspects of senescence, rather than immediately performing gene-array studies. Future studies will require the use of RNA-seq as an unbiased approach to using tissues from different gestational stages and then test known genes, as well as novel genes, to further support our initial findings. Our data demonstrate that placental oxidative stress and senescence increase, in parallel with a reduction in PlGF expression, as normotensive pregnancy progresses, while antioxidant defences diminish as gestational age increases. These features become evident earlier in gestation in women with pre-eclampsia, suggesting accelerated senescence, probably secondary to their poor antioxidant status [13]. If these markers can be detected in the circulation, they may prove useful in screening for women with more severe problems of post-term pregnancy and pre-eclampsia.

## 4. Materials and Methods

### 4.1. Cohort and Sample Collection

The study population (Table 1) consisted of two groups of normotensive women: term (*n* = 26), with gestational age of 37–40^+6^ weeks, and post-term (*n* = 11), with gestational age of 41–42 weeks. The inclusion criteria comprised no maternal or pregnancy complications, live birth, singleton pregnancy, and delivery either vaginally or by Caesarean section. A third group consisted of women diagnosed with pre-eclampsia (*n* = 13) and delivered between 31^+2^ and 41^+2^ weeks. Pre-eclampsia was defined as systolic blood pressure of ≥140/90 mmHg on two occasions and proteinuria ≥300 mg/L, 500 mg/day, or ≥2+ on a dipstick analysis of midstream urine after 20 weeks [36]. Detailed demographics and outcome data have previously been published [37]. The study was approved by the HRA-REC ethics committee of the University of Nottingham (REF: 15/EM/0523); written, informed consent to take part in the study was obtained from each participant.

Full-depth tissue biopsies were collected within 10 min of the placenta being delivered as previously described [37]. Samples were taken from the mid-point between the umbilical cord insertion and the periphery of the placenta, avoiding infarcts. One set of samples was snap-frozen and stored at −80 °C for RNA analysis. The other set was formalin-fixed and embedded in paraffin wax for immunohistochemistry.

### 4.2. RNA Extraction, cDNA Synthesis, and Quantitative Reverse-Transcription Polymerase Chain Reaction (RT-qPCR)

Total RNA was extracted from ~100 mg of placental tissue using QIAzol lysis reagent (Qiagen, UK) as previously described [38]. RNA (1 μg) was reverse-transcribed using the QuantiTect Reverse Transcription kit (Qiagen, UK) in a Primus96 thermocycler (Peqlab Ltd., Southampton, UK). RT-qPCR was carried out using SYBR Green chemistry (2× QuantiFast SYBR Green, Qiagen, UK) on an AB7500 Fast (Life Technologies, Cramlington, UK) using primers to *NADPH oxidase 4 (NOX4), glutathione peroxidase 1, 3,* and *4 (GPX1, GPX3 GPX4), cyclin-dependent kinase inhibitor 2A (P16^INK4α^), cyclin-dependent kinase inhibitor (P21), interleukin-6 (IL-6), placental growth factor (PlGF)*, and *soluble fms-like tyrosine kinase-1 (a splice variant of VEGF receptor 1, sFlt-1*; Table 2). Cycling conditions were as follows: a pre-PCR cycle was run for 15 min at 95 °C followed by 40 cycles of 95 °C for 10 s and 60 °C for 30 s. Abundance data for the genes of interest were expressed as normalised copy number following normalisation using GeNORM (http://medgen.ugent.be/~jvdesomp/genorm/; accessed on 3 November 2018), with stably expressed reference genes [39] *beta-2 microglobulin (B2M), tyrosine 3-monooxygenase/tryptophan 5-monooxygenase activation protein zeta (YWHAZ)*, and *glyceraldehyde 3-phosphate dehydrogenase (GADPH)* (Table 2).

### 4.3. Immunohistochemical Staining

Placental protein expression was assessed by immunohistochemistry as previously described [38], using antibodies to NOX4, 8-Oxo-2′-deoxyguanosine (8-OHdg), P16^INK4α^, IL-6, and PlGF at concentrations detailed in Table 3. Immunoglobulin G (IgG) from the same host as the primary antibody was used as a negative control. All slides were assessed by the same observer, blinded to group. Quantification was performed as described previously [38], using the Positive Pixel Algorithm of Aperio ImageScope software; a visual check was also performed.

### 4.4. Statistical Analysis

All tests were performed using SPSS version 26 and GraphPad Prism version 8. Summary data are presented as means ± standard deviation (SD) or median and interquartile range (IQR) as appropriate. The Kruskal–Wallis test, followed by Mann–Whitney U-test, was used for multiple group analysis. Student’s *t*-tests or Mann–Whitney U-tests were applied depending on whether the data distribution was normal or skewed, as indicated by the Kolmogorov–Smirnov test. The null hypothesis was rejected when *p* < 0.05.

## Figures and Tables

**Table 1:** Participant demographics and pregnancy outcome data.

Parameter	Term 37–39^+6^ (*n* = 26)	Post-Term 41–42 (*n* = 11)	Pre-Eclampsia (*n* = 13)
Age at booking (year)	31 ± 6.0	31 ± 6.4	34 ± 3.8
BMI at booking (kg/m^2^)	26 [23,32]	27 [26,31]	30 [26,31]
Nulliparous, *n* (%)	10 (38)	5 (45)	8 (62)
Smoking, *n* (%)	5 (19)	3 (27)	1 (8)
Systolic blood pressure (mmHg)	110 ± 10.6 ^c^	118 ± 5.1 ^c^	158 ± 12.3 ^c^
Diastolic blood pressure (mmHg)	77 ± 7.8 ^c^	75 ± 6.0 ^c^	97 ± 6.8 ^c^
Proteinuria	-	-	1.0 [0.4, 1.4]
Gestational age at delivery (weeks)	39.4 (36.3–40.6) ^a,c^	41.3 (41.0–42) ^a,b^	37.2 (31.1–41.1) ^b,c^
Birth weight (g)	3533 ± 378 ^a^	3873 ± 384 ^b^	2741 ± 1073.7 ^a,b^
Birthweight centile	74 [47,94]	86 [65,94]	48 [26,78]
Placental weight (g)	624 ± 133.6	696 ± 92.6 ^b^	539 ± 182.9 ^b^
Baby gender, female *n* (%)	14 (54)	5 (45)	8 (62)
Caesarean Section *n* (%)	19 (73)	11 (73)	8 (77)

^a b c^ *p* < 0.05 between the respective groups. Data are presented as mean ± SD, median [IQR], or median (range) for gestational age at delivery, depending on distribution or *n* (%). BMI: body mass index. Birthweight centiles calculated using INTERGROWTH 21 (https://intergrowth21.tghn.org/standards-tools/; accessed on 25 May 2021).

**Table 2:** Details of primers used.

Gene	Accession Number	Primers	Length (bp)
*Nox4*	NM_016931.3	5′–TGAACTATGAGGTCAGCCTCTG–3′ 5′–TCTCACGAATCTCCTCATGGT–3′	107
*GPx1*	NM_201397	5′–CAGTCGGTGTATGCCTTCTCG–3′ 5′–GAGGGACGCCACATTCTCG–3′	105
*GPx3*	NM_002084	5′–GAGCTTGCACCATTCGGTCT–3′ 5′–GGGTAGGAAGGATCTCTGAGTTC–3′	94
*GPx4*	NM_001039847	5′–GAGGCAAGACCGAAGTAAACTAC–3′ 5′–CCGAACTGGTTACACGGGAA–3′	100
*P16^INK4a^*	NM_000077.4	5′–CTTCGGCTGACTGGCTGG–3′ 5′–TCATCATGACCTGGATCGGC–3′	129
*P21*	NM_078467	5′–TGTCCGTCAGAACCCATGC–3′ 5′–AAAGTCGAAGTTCCATCGCTC–3′	139
*IL–6*	NM_000600	5′–ACTCACCTCTTCAGAACGAATTG–3′ 5′–CCATCTTTGGAAGGTTCAGGTTG–3′	149
*PlGF*	NM_001207012	5′–GAACGGCTCGTCAGAGGTG–3′ 5′–ACAGTGCAGATTCTCATCGCC–3′	187
*sFlt–1*	NM_001159920	5′–TTTGCCTGAAATGGTGAGTAAGG–3′ 5′–TGGTTTGCTTGAGCTGTGTTC–3′	117
*B2M*	NM_004048.2	5′–CTTATGCACGCTTAACTATCTTAACAA–3′ 5′–TAGGAGGGCTGGCAACTTAG–3′	127
*YWHAZ*	NM_001135702.1	5′–ACTTTTGGTACATTGTGGCTTCAA–3′ 5′–CCGCCAGGACAAACCAGTAT–3′	94
*GAPDH*	NM_002046.3	5′–GGAAGCTTGTCATCAATGGAA–3′ 5′–TGGACTCCACGACGTACTCA–3′	102

**Table 3:** Antibody details.

Antigen	Suppler Information	Concentration (µg/mL)
NOX4	Abcam, rabbit monoclonal: ab133303	2.18
8-0HdG	Abcam, mouse monoclonal: ab48508	12
p16^INK4α^	Abcam, rabbit polyclonal: ab108349	0.7
IL-6	Abcam, mouse monoclonal: ab9324	
PlGF	Abcam, rabbit polyclonal: ab196666	10

## Data Availability

Data is contained within the article and additional raw data can be obtained from the corresponding author on request.
